# Extracellular vesicle-cargo miR-185-5p reflects type II alveolar cell death after oxidative stress

**DOI:** 10.1038/s41420-020-00317-8

**Published:** 2020-09-10

**Authors:** Jonathan M. Carnino, Heedoo Lee, Xue He, Michael Groot, Yang Jin

**Affiliations:** Division of Pulmonary and Critical Care Medicine, Department of Medicine, Boston University, Boston, MA 02118 USA

**Keywords:** Respiratory distress syndrome, Translational research, Apoptosis

## Abstract

Acute respiratory distress syndrome (ARDS) is a devastating syndrome responsible for significant morbidity and mortality. Diffuse alveolar epithelial cell death, including but not limited to apoptosis and necroptosis, is one of the hallmarks of ARDS. Currently, no detectable markers can reflect this feature of ARDS. Hyperoxia-induced lung injury is a well-established murine model that mimics human ARDS. We found that hyperoxia and its derivative, reactive oxygen species (ROS), upregulate miR-185-5p, but not miR-185-3p, in alveolar cells. This observation is particularly more significant in alveolar type II (ATII) than alveolar type I (ATI) cells. Functionally, miR-185-5p promotes expression and activation of both receptor-interacting kinase I (RIPK1) and receptor-interacting kinase III (RIPK3), leading to phosphorylation of mixed lineage kinase domain-like (MLKL) and necroptosis. MiR-185-5p regulates this process probably via suppressing FADD and caspase-8 which are both necroptosis inhibitors. Furthermore, miR-185-5p also promotes intrinsic apoptosis, reflected by enhancing caspase-3/7 and 9 activity. Importantly, extracellular vesicle (EV)-containing miR-185-5p, but not free miR-185-5p, is detectable and significantly elevated after hyperoxia-induced cell death, both in vitro and in vivo. Collectively, hyperoxia-induced miR-185-5p regulates both necroptosis and apoptosis in ATII cells. The extracellular level of EV-cargo miR-185-5p is elevated in the setting of profound epithelial cell death.

## Introduction

Acute respiratory distress syndrome (ARDS) is a devastating syndrome responsible for significant morbidity and mortality. Its mild form was formerly named acute lung injury (ALI). Non-cardiogenic pulmonary edema, vascular leakage, inflammation, lung epithelial cell injury, and dysfunction are key features of ARDS/ALI. Despite recent progress in protective ventilation strategies, the fundamental pathogenesis of ALI/ARDS remains poorly understood, impeding the development of highly effective prevention and/or treatment strategies.

Hyperoxia-induced lung injury (HALI) is a well-established murine model that mimics human ARDS, which has been extensively used to better understand the pathogenesis of ARDS^1^. One of the prominent features of ARDS is alveolar epithelial cell death^2^. Hyperoxia is known to induce lung epithelial cell death by mechanisms such as necrosis, apoptosis, necroptosis, and autophagic cell death^3^. Currently, the molecular mechanisms behind hyperoxia-induced alveolar cell death remains incompletely understood, requiring further investigation. Furthermore, there is no detectable markers which reflect the undergoing alveolar cell death, thus missing an opportunity to develop early diagnostics and therapeutics.

Common mechanisms of cell death, which are most regularly studied now, are apoptosis, necrosis, necroptosis, and pyroptosis. Programmed cell death, or apoptosis, normally occurs during phases of development and aging, as a mechanism to regulate cell populations within tissues^4^. Apoptosis is a known cellular response to stimuli that is slightly below the threshold which would induce necrosis, such as heat, toxins, trauma, and membrane peroxidizing reagents to name a few^5^. Apoptotic cells are characterized by chromatin condensation, nuclear fragmentation, plasma membrane budding, and cell shrinkage^6^. Necrosis is characterized by swelling of the cell, enlargement of various cellular organelles, random degradation of DNA, substantial plasma membrane endocytosis, and autophagy^7^. Necrosis is usually triggered by either extreme environmental conditions or genetically encoded defects^8^. Since necrosis does not require new protein synthesis, has very minimal energy requirements, and is not a homeostatically regulated mechanism, it is generally understood as a passive process^9^. Necroptosis is well understood as regulated necrosis, involving RIPK1, RIPK3, and MLKL^10^. Multiple studies have reported that inhibition of caspase-8 can shift a cell from apoptosis to a necrosis form of cell death by the latter activation of RIPK3 and MLKL, leading to necroptosis^11^. It has been reported that inhibition of caspase-8 molecules results in activation of RIPK3, which is a key role in necroptosis^12^. The ultimate activation of MLKL through phosphorylation leads to translocation of the phospho-protein into the inner leaflet of the plasma membrane, which disrupts the cell’s integrity^13^.

The cross talk between necroptosis and apoptosis is well established but still not fully understood. It has been determined that there is modulation between different cell death processes by mutual inhibitory mechanisms, which work as a substitute cell death route in the case of a defect in the primary route^14^. One example of this cross-talk between necroptosis and apoptosis is that RIPK1 is known to protect cells from caspase-8 apoptosis by contributing to the induction of antiapoptotic genes^14^. In addition, active caspase-8, which is known to be involved in apoptosis, inactivates the necroptosis mediators such as RIPK1 and RIPK3^15^. Recent reports show that cross-talk between different cell death mechanisms can also be controlled by microRNAs (miRNAs).

miRNAs are small, single-stranded non-coding RNAs (ncRNAs) that function to regulate gene expression. MiRNAs are first transcribed by RNA polymerase to generate precursor miRNAs, which then undergo a two-step cleavage process before becoming mature miRNA that are incorporated into the RNA-induced silencing complex (RISC)^16^. RISC then uses these mature miRNAs to bind to messenger RNA (mRNA) via direct sequence-specific binding^17^. Via this mechanism, these miRNA guided RISC complexes target specific genes for degradation or translational inhibition^18^. Thus, miRNAs are able to regulate the expression of target mRNAs and proteins within a cell, a mechanism that plays a crucial role in the pathogenesis of many diseases. MiR-155,-128a, and miR-512-3p are some of the known miRNAs which regulate necroptosis, whereas there are many more known miRNAs which regulate apoptosis—including miR-224, −21, −103, −125b, −504, and miR-373 to name some^19^. The cross talk between necroptosis and apoptosis can be controlled by miR-874, which reportedly enhances necroptosis activation by targeting caspase-8, a key gene in the transition from apoptosis to necroptosis^19^.

We previously reported that hyperoxia-induced miR-185 contributed to epithelial cell death via enhanced DNA damage and modulation of 14-3-3δ pathways^20^. Our previous studies did not clarify the type of alveolar cells which are regulated by miR-185. Furthermore, whether miR-185-3p or -185-5p is responsible for the regulation of hyperoxia-induced alveolar cell death was still unclear. In the precursor miRNA structure, the 5p strand is present in the forward (5′-3′) position and the 3p strand (complementary to the 5p strand) is located in the reverse position. Following Dicer cleavage of the precursor stem loop, either the 5p or 3p strand can be functional. However, the more stable strand will play a more important role functionally while the less stable strand will often be degraded. To serve as a potential therapeutic target, it is essential to clearly understand which strand plays the major role in a given pathophysiological process.

To understand its cellular importance and its validity as a possible therapeutic target, it is crucial to thoroughly study the detailed mechanism by which miR-185 regulates cell death. Cell death is a complicated process and each type of cell death may play the major role at different time points or under different exposure dosages. Furthermore, all types of cell death may co-exist or overlap at a given time point and may be regulated by the same factor, or each type of cell death may have its own specific regulator. Although alveolar cell death is a hallmark of the early phases of ARDS, there is no diagnostic marker available at this time.

Extracellular vesicles (EVs) are small, lipid-bound vesicles excreted from cells that contain protein and nucleic acid cargo^21^. The International Society for Extracellular Vesicles (ISEV), classifies EVs into three major categories, apoptotic bodies (ABs), microvesicles (MVs), and exosomes (Exos), based on their size, surface markers, and method of generation^22^. It is well established that during apoptosis cells release vesicles into the extracellular compartment, however, recently it has been discovered that healthy cells also release EVs^21^. EVs can be isolated from most cell types, and are found in biological fluids as well, which includes bronchial-alveolar-lavage fluid (BALF)^21^. Similar to miRNAs being used as biomarkers of disease or illness, EV’s hold the same potential. Further investigation is required to understand their potential functions and uncover whether EV-cargos serve as potential cell-specific biomarkers.

This work focuses on the functional role of miR-185-5p in the development of necroptosis and potential crosstalk between necroptosis and apoptosis. Our current study further examines the level of EV-cargo miR-185-5p in serum after hyperoxia-induced alveolar epithelial cell death.

## Results

### MiR-185-5p and miR-185-3p are differentially induced in lung epithelial cells after exposure to hyperoxia

**Fig. 1:**
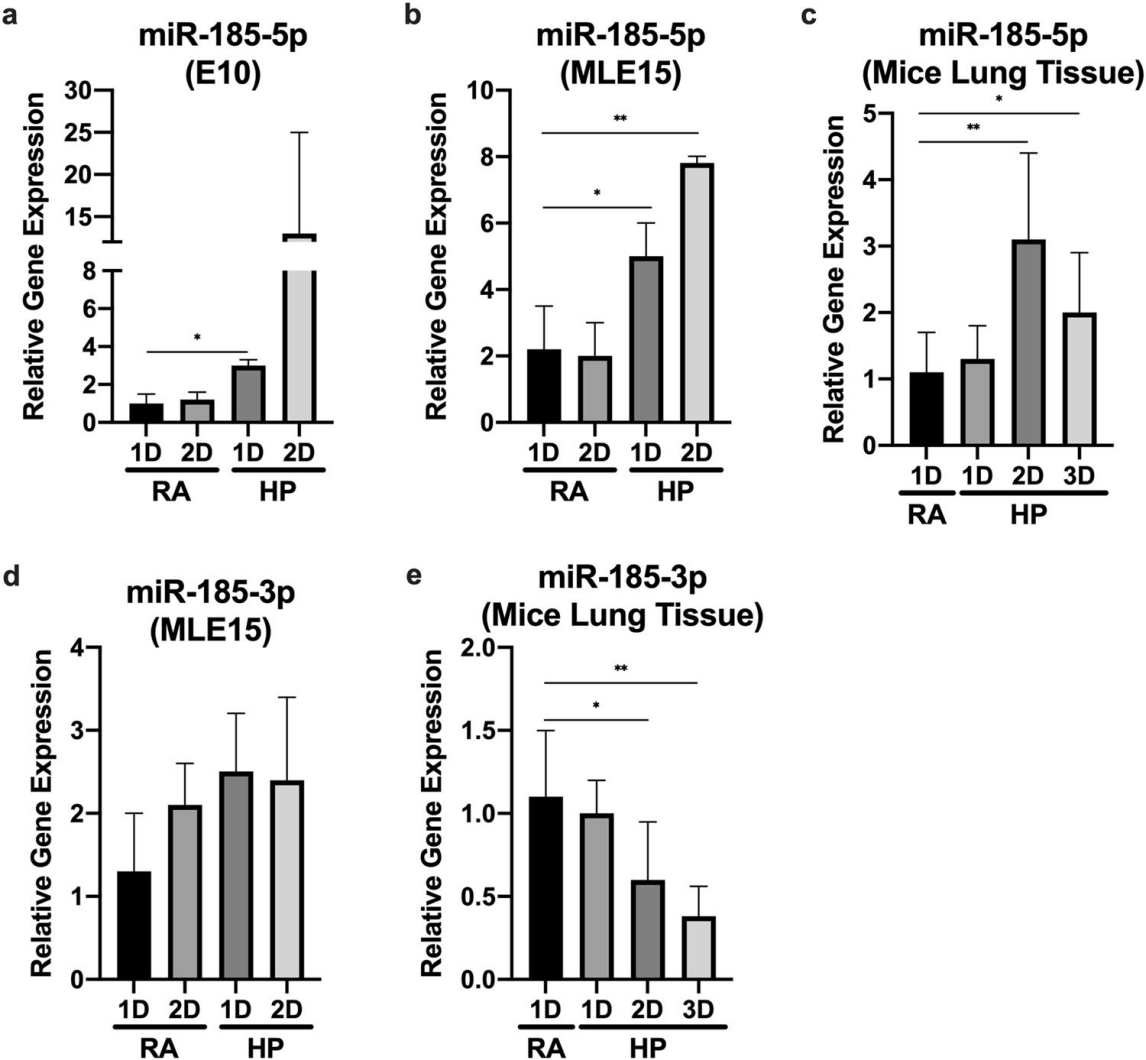
Effects of hyperoxia on miR-185-5p and miR-185-3p expression levels in epithelial cells and lung tissue. **a**–**c** Time course of hyperoxia-induced expression of miR-185-5p in (**a**) E10, (**b**) MLE15, and (**c**) mice alveoli tissue. **d**, **e** Time course of hyperoxia-induced expression of miR-185-3p in (**d**) MLE15 and (**e**) mice alveoli tissue. The expression level was measured using real-time PCR. The figures in (**a**, **b**, **d**) represent three independent experiments (*n* = 4 samples per group) with identical results. The figures in panels c and e represent three independent experiments (*n* = 4 mice per group) with identical results. Mean ± SD; *p < 0.05, **p < 0.01.

Our previous report found that miR-185 expression increased in both Beas2B cells and mice lung epithelial cells after oxidative stress^20^. To determine whether miR-185-3p or 5p is more important, we first subjected wild-type (WT) C57BL/6 mice, mouse alveolar type II cells (MLE15), and mouse alveolar type I cells (E10) to hyperoxia (100% oxygen) treatment. We found that hyperoxia induces miR-185-5p expression in both E10 and MLE15 cells in a time-dependent manner, but expression increased most significantly in MLE15 cells (Fig. 1a, b). Next, we confirmed that miR-185-5p was also induced in mice lung tissue with hyperoxia treatment (Fig. 1c). In addition to checking miR-185-5p, expression levels of miR-185-3p were also checked in MLE15 cells, but there was no significant induction found (Fig. 1d). Expression levels of miR-185-3p after treatment of hyperoxia was also analyzed in mouse lung tissue. Interestingly, we found a decrease in miR-185-3p level in a time-dependent manner (Fig. 1e).

### MiRNA-185-5p regulates the expression of RIPK1 and RIPK3

**Fig. 2:**
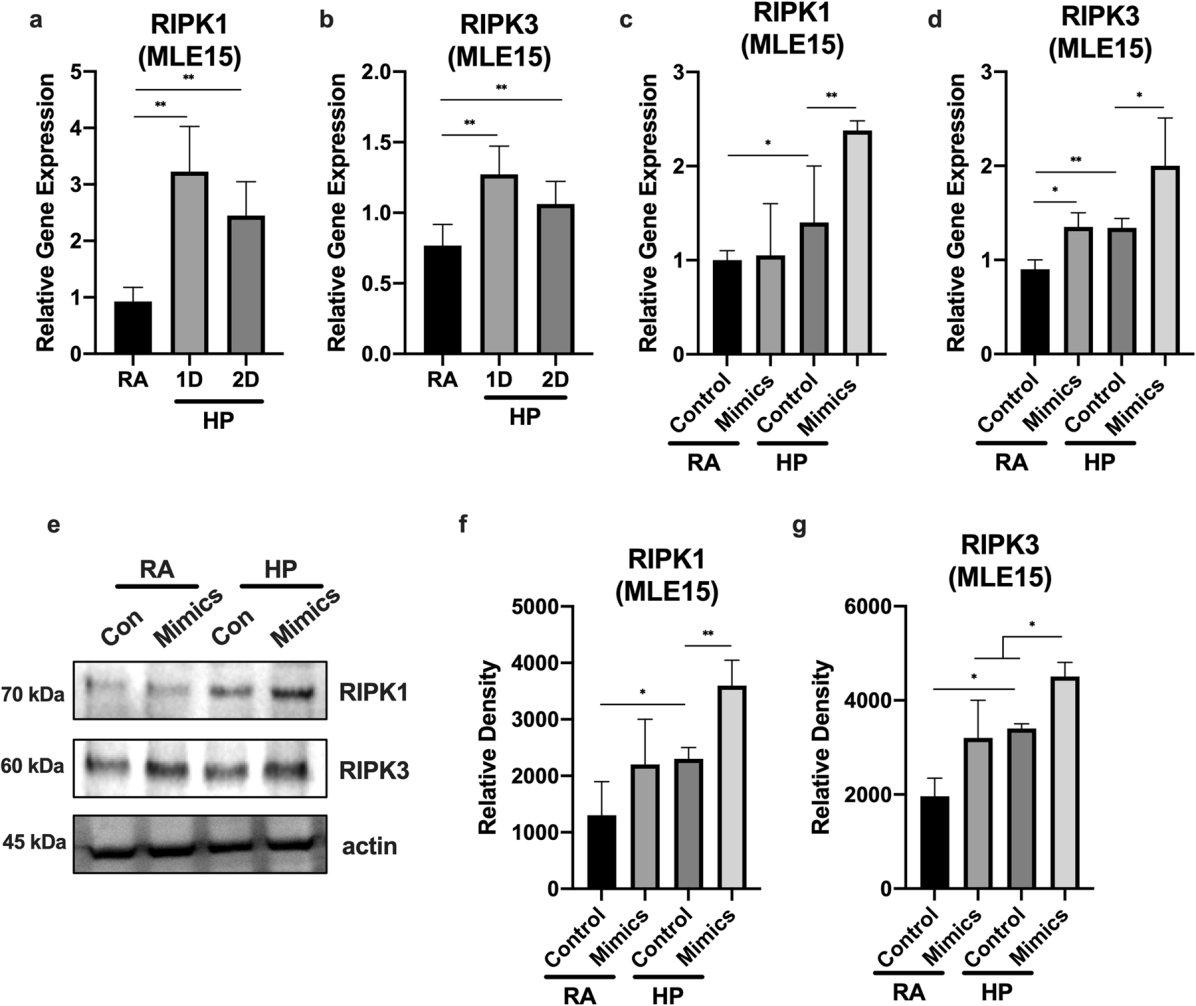
MiR-185-5p aggravated the hyperoxia-induced necroptosis gene markers in lung epithelial cells. **a**, **b** MLE15 cells were treated with room air or hyperoxia for 1 and 2 days. **a** RIPK1 gene expression and **b** RIPK3 gene expression level was measured using real-time PCR. **c**, **d** MLE15 cells were transfected with miRNA-185-5p mimics, and treated with hyperoxia for 1 day. **c** RIPK1 gene expression and **d** RIPK3 gene expression level was measured using real-time PCR. **e** MLE15 cells were treated with room air or hyperoxia for 1 day and RIPK1 and RIPK3 protein expression was measured by Western blot. **f**, **g** Protein levels were quantified and normalized to actin. The figures represent three independent experiments (*n* = 4 samples per group) with identical results. Mean ± SD; *p < 0.05, **p < 0.01.

Based on previously published reports, both RIPK1 and RIPK3 are protein markers of necroptosis^23^. To determine whether hyperoxia alters RIPK1/RIPK3 level in lung epithelial cells, we check RIPK1 and RIPK1 protein levels after treatment with hyperoxia (24 h). We found that treatment of MLE15 cells with hyperoxia leads to the induction of both RIPK1 and RIPK3 gene expression, detected by real-time RT-PCR (Fig. 2a, b). This result suggests that hyperoxia regulates RIPK1/RIPK3 expression at the transcriptional level. MiRNAs are well known to regulate target gene expression transcriptionally^18^. To check if miRNA-185-5p played a role in the regulation of the RIPK1/RIPK3 proteins, we used “gain-of-function” approaches. MLE15 cells transfected with miR-185-5p mimics (0.038nmol for 24 h) showed an increase in relative gene expression when compared to cells treated with control mimics (Fig. 2c, d). In addition, treatment of MLE15 cells with miR-185-5p mimics lead to an increase in protein expression compared to cells treated with control mimics (Fig. 2e–g).

### Reactive oxygen species (ROS) regulate miR-185-5p levels in MLE15 cells

**Fig. 3:**
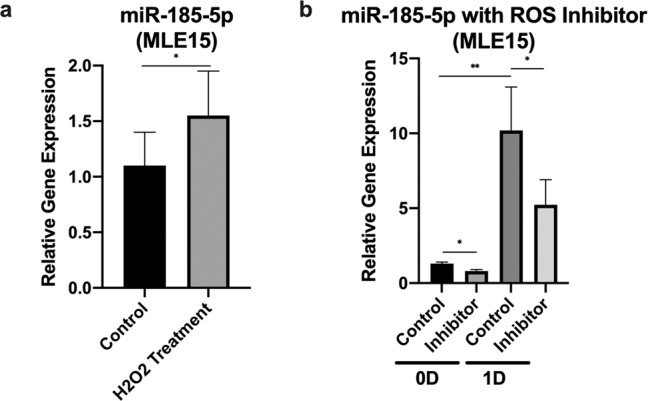
ROS plays a role on miR-185-5p levels in MLE15 cells. **a** MLE15 cells were exposed to 90 μM H_2_O_2_ for 5 h. **b** MLE15 cells were exposed to room air or hyperoxia 1 day in the presence or absence of 3 mM of N-acetyl-l-cysteine (NAC). The expression level was measured using real-time PCR. The figures represent three independent experiments (*n* = 3 samples per group) with identical results. Mean ± SD; *p < 0.05, **p < 0.01.

Hyperoxia-induced cell death is well known to function through the generation of ROS^24^. To determine if ROS played a role in miR-185-5p induction with hyperoxia, we first checked the expression level of miR-185-5p with the treatment of H_2_O_2_ (a well-documented ROS generator). We found a strong induction of miR-185-5p in MLE15 cells with the treatment of H_2_O_2_ (Fig. 3a). To further confirm the role of ROS in hyperoxia-induced miR-185-5p expression, we pre-treated MLE15 cells with 5 mM NAC for 30 min, followed by hyperoxia for 24 h. We confirmed that NAC pre-treatment blocked the hyperoxia-induced miR-185-5p expression in MLE15 cells (Fig. 3b).

### MiR-185-5p regulates the downstream phosphorylation of MLKL and hyperoxia induces necroptosis marker protein RIPK1/RIPK3 via ROS

**Fig. 4:**
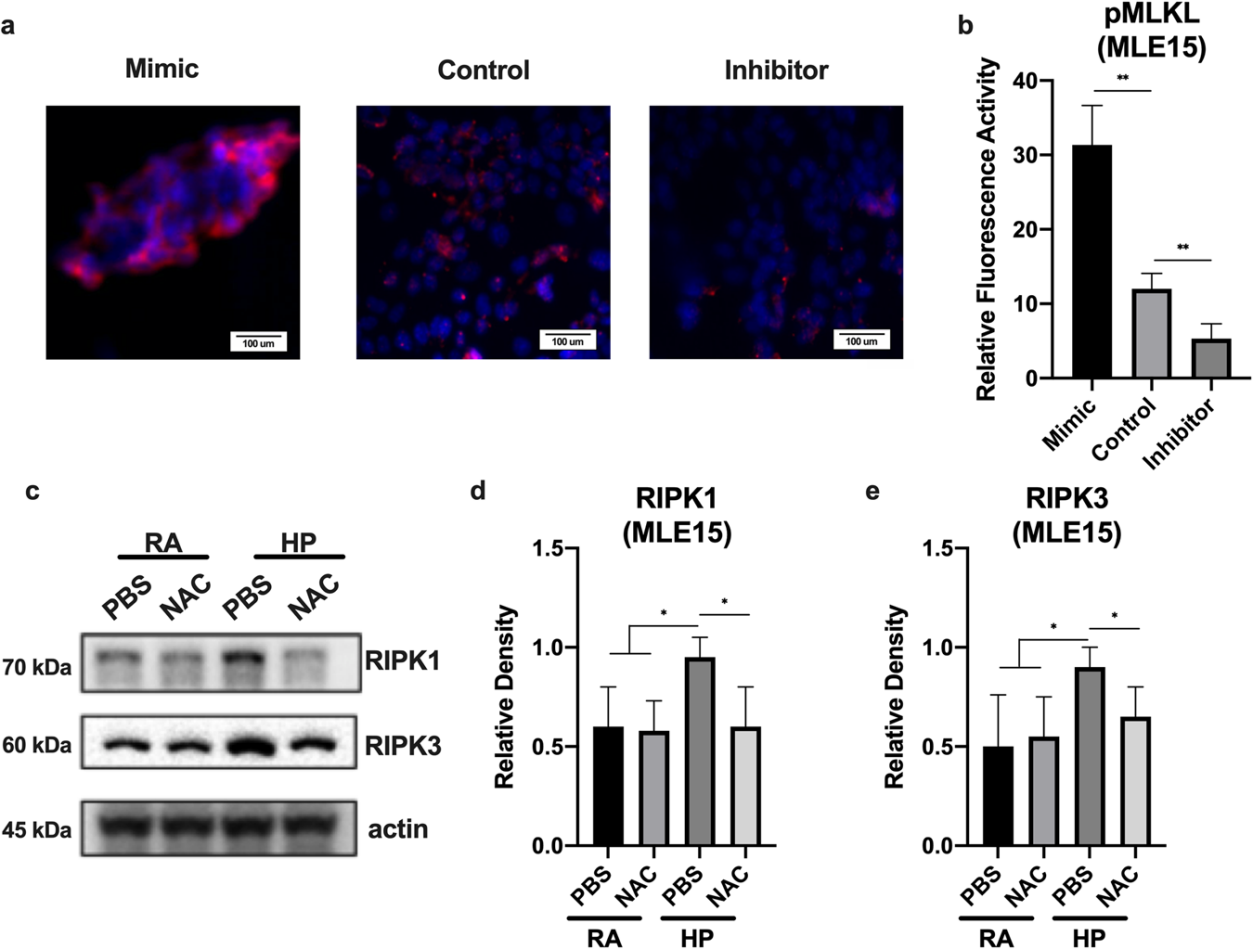
Necroptotic protein marker expression was altered by miR-185 mimics and inhibitors. Expression of necroptosis gene markers after hyperoxia is mediated by ROS. **a**, **b** MLE15 cells were transfected with miRNA-185-5p inhibitors, and treated with hyperoxia for 1 day. **a** Immunofluorescence analysis of phospho-MLKL. **b** Quantified immunofluorescence analysis of relative protein expression. **c** MLE15 cells were exposed to room air or hyperoxia 1 day in the presence or absence of 3 mM of N-acetyl-l-cysteine (NAC), and RIPK1 and RIPK3 protein expression was measured by Western blot. **d**, **e** Protein levels were quantified and normalized to actin. The figures represent three independent experiments (*n* = 4 samples per group) with identical results. Mean ± SD; *p < 0.05, **p < 0.01.

Published reports have indicated that the phosphorylation of MLKL by RIPK3 is the final step in a cascade triggering necroptosis^13,23,25^. To investigate whether miR-185-5p played an upstream regulatory role on necroptosis, we used the “loss-of-function” approaches. Using immunofluorescence, we found MLE15 cells transfected with miR-185-5p mimics (0.04 nmol for 24 h) expressed an increased level in relative phospho-MLKL expression when compared to cells treated with control mimics; Consistently, MLE15 cells transfected with miR-185-5p inhibitors (0.04 nmol for 24 h) showed a decrease in relative phospho-MLKL expression (Fig. 4a, b). To determine whether hyperoxia alters RIPK1/RIPK3 level in lung epithelial cells, we checked RIPK1 and RIPK1 protein levels after treatment with hyperoxia (24 h) in the presence and absence of NAC (5 mM NAC for 30 min). We also confirmed that the treatment of MLE15 cells with NAC and hyperoxia inhibited the expression of RIPK1/RIPK3 at the protein level (Fig. 4c–e).

### Effects of miR-185-5p on apoptosis and caspase-8 expression

**Fig. 5:**
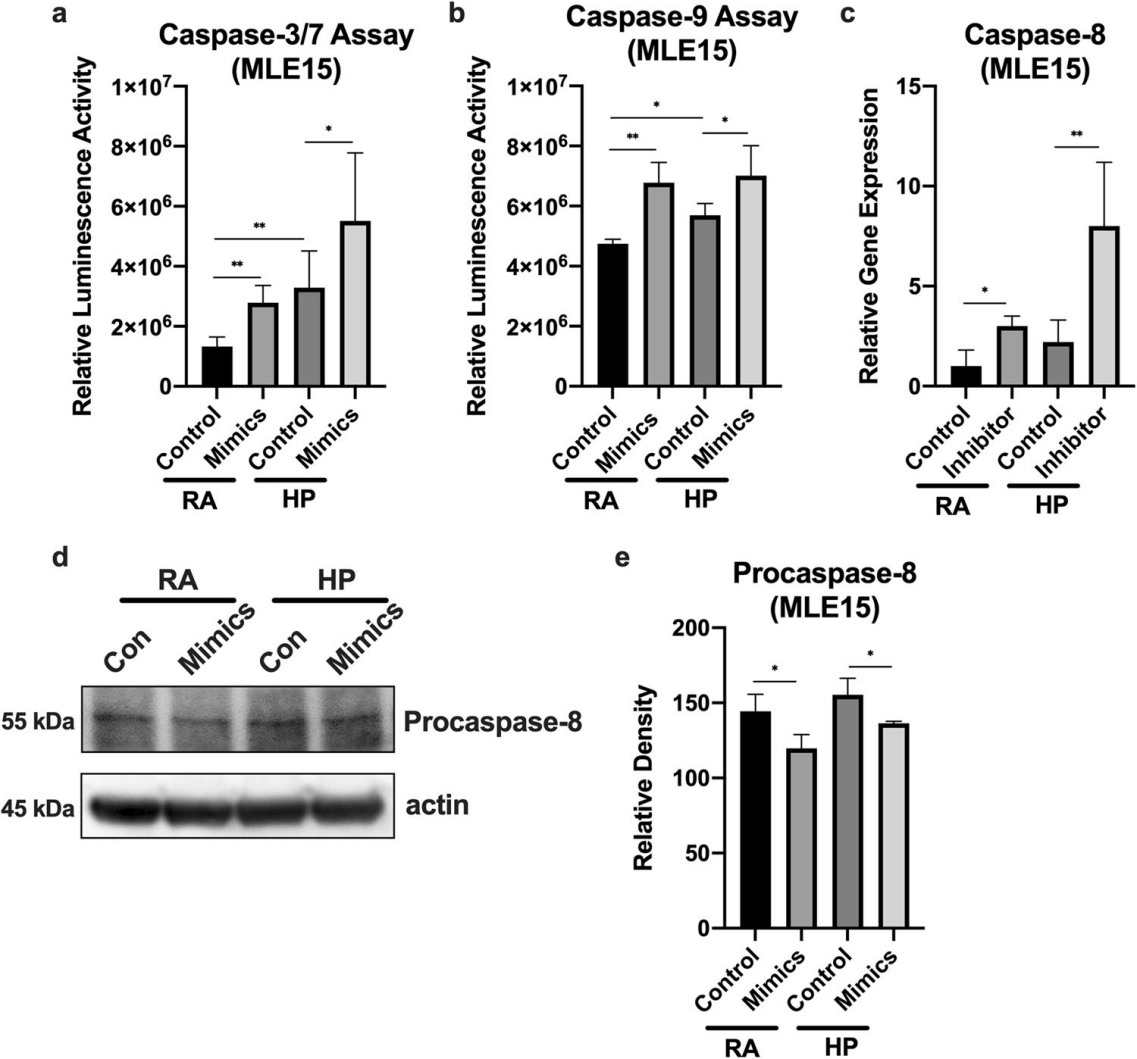
Effects of miR-185-5p on hyperoxia-induced apoptosis and caspase-8 expression. **a**, **b** MLE15 cells were transfected with miR-185-5p mimics and treated with room air or hyperoxia for 1 day, then (**a**) relative caspase-3/7 and (**b**) relative caspase-9 enzyme activity was measured. **c** MLE15 cells were exposed to hyperoxia for 1 day in the presence or absence of miR-185-5p inhibitor, and caspase-8 gene expression level was measured using real-time PCR. **d**, **e** MLE15 cells were transfected with miR-185-5p mimics and treated with room air or hyperoxia for 1 day, then (**d**) procaspase-8 protein expression was measured by Western blot. **e** Protein levels were quantified and normalized to actin. The figures represent four independent experiments (*n* = 4 samples per group) with identical results. Mean ± SD; *p < 0.05, **p < 0.01.

Caspase-3, −7, and −9 are known to play roles during intrinsic apoptosis^26^. To test for apoptosis regulation by miR-185-5p, we used “gain-of-function” approaches which showed both caspase-3/7 and caspase-9 activity was induced by miR-185-5p mimics (Fig. 5a, b). In addition, it is understood that the activation of FADD leads to the recruitment and activation of caspase-8-associated extrinsic apoptotic pathway^27,28^. To determine whether miR-185-5p regulated the FADD/caspase-8 complex, which prevents necroptosis by blocking RIPK1 and RIPK3, we first checked caspase-8 gene expression using the “loss-of-function” approach. Caspase-8 gene expression was significantly induced by the inhibition of miR-185-5p in MLE15 cells treated with room air or hyperoxia (Fig. 5c). Using the “gain-of-function” approach, we then checked procaspase-8 protein expression level using Western blot analysis. We found that miR-185-5p mimics inhibited the protein expression of procaspase-8, further confirming the regulation of caspase-8 by miR-185-5p (Fig. 5d, e).

### Effects of miR-185-5p on necroptosis/apoptosis cross-talk in alveolar epithelial type II cells

#### FADD is a direct target of miR-185-5p

**Fig. 6:**
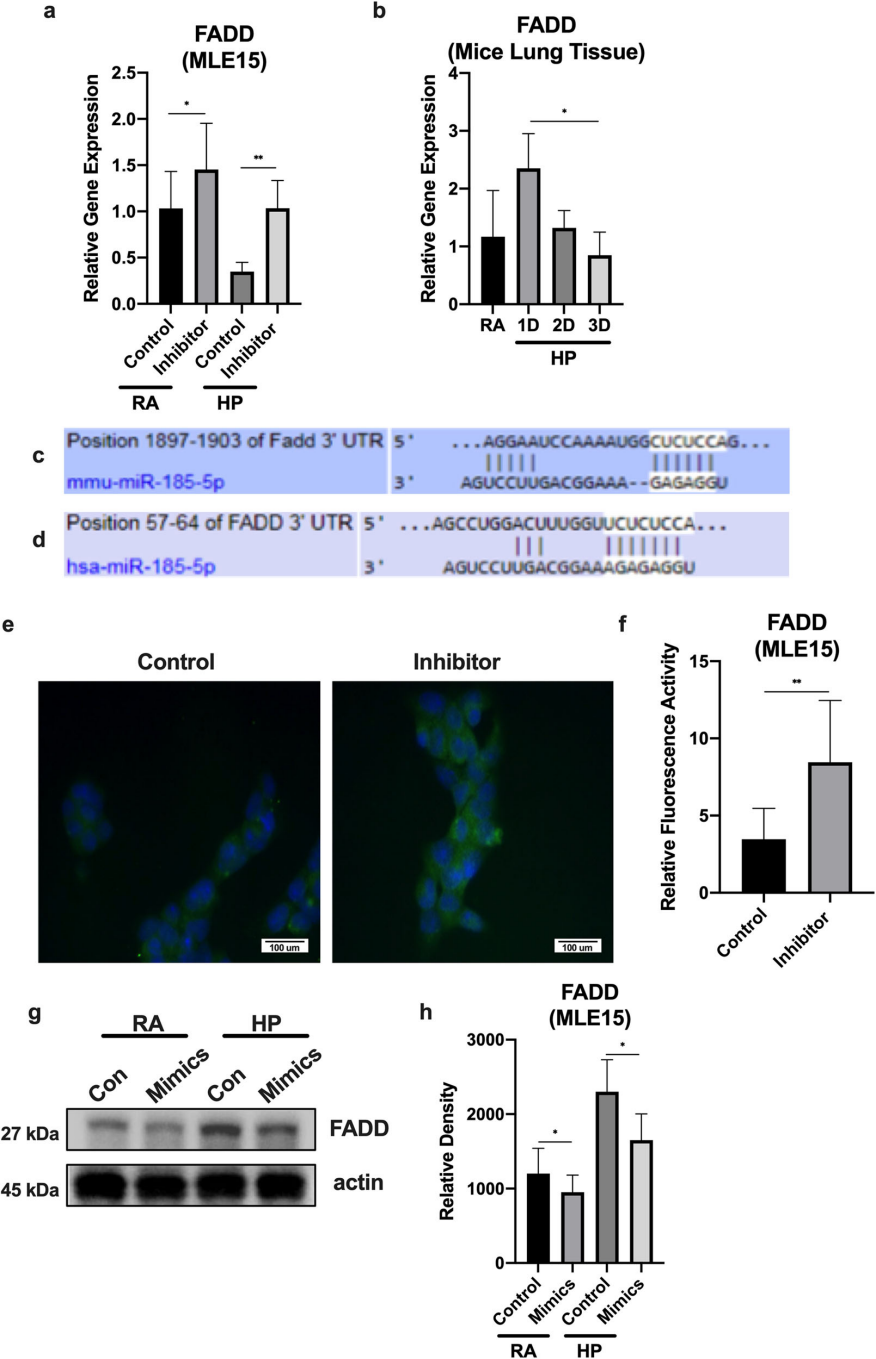
miR-185-5p suppresses FADD protein and mRNA expression levels. **a** MLE15 cells were exposed to hyperoxia for 1 day in the presence or absence of miR-185-5p inhibitor. The expression level was measured using real-time PCR. **b** Live mice were treated with hyperoxia for 0 days, 1 day, 2 days, and 3 days, and RNA was isolated from alveoli tissue. The expression level was measured using real-time PCR. **c**, **d** FADD is a direct target of miR-185-5p in both (**c**) mice and (**d**) humans. **e**, **f** MLE15 cells were transfected with either miR-185-5p mimics or inhibitors, and treated with room air or hyperoxia for 1 day. **e** Immunofluorescence analysis of phospho-MLKL **f** Quantified immunofluorescence analysis of relative protein expression. **g** and **h**: MLE15 cells were transfected with miR-185-5p mimics and treated with room air or hyperoxia for 1 day, then (**d**) FADD protein expression was measured by Western blot. **e** Protein levels were quantified and normalized to actin. The figures represent four independent experiments (*n* = 4 samples per group) with identical results. Mean ± SD; *p < 0.05, **p < 0.01.

Past studies have uncovered that the activation of FADD and caspase-8 forms a complex which inhibits the activation of RIPK1 and RIPK3 in necroptosis^27,29^. First, we found that the treatment of hyperoxia caused a decrease in the expression of FADD in mice tissue (Fig. 6b). Consistently, hyperoxia decreased the gene expression of FADD in MLE15 cells. Next, we found that inhibition of miR-185-5p attenuated the effects of hyperoxia on FADD expression (Fig. 6a). Based on *Targetscan.com*, we predicted that FADD is a potential target of miR-185-5p in both mice and humans (Fig. 6c, d). To further confirm the effects of miR-185-5p on FADD expression, we evaluated its protein level using a “loss-of-function” approach. Using immunofluorescence, we confirmed that there was an increase in FADD protein levels when MLE15 cells were transfected with miR-185-5p inhibitors (0.04 nmol for 24 h) (Fig. 6e, f). To further confirm these findings, using the “gain-of-function” approach, we then checked FADD protein expression level using Western blot analysis. We found that miR-185-5p mimics inhibited the protein expression of FADD, further confirming the regulation of FADD by miR-185-5p (Fig. 6g, h).

### MiR-185-5p is secreted by ATII cells via EVs and its level in EVs is elevated after hyperoxic cell death

**Fig. 7:**
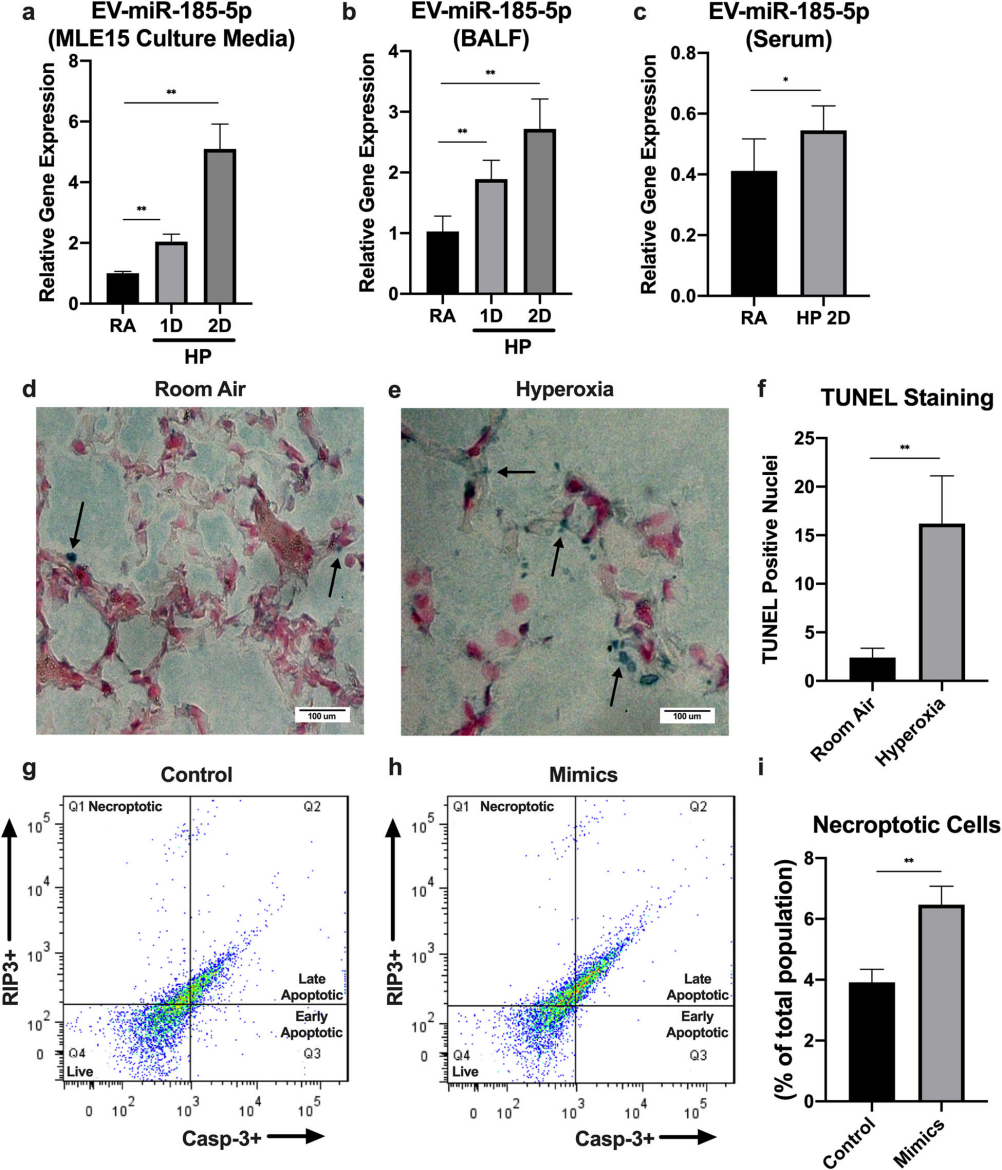
EV-cargo miR-185-5p correlates the cell death of lung epithelial cells. **a**–**c** Time course of hyperoxia-induced expression of EV-miR-185-5p in (**a**) MLE15 cell-culture media, (**b**) BALF, and (**c**) serum. The expression level was measured using real-time PCR. **d**–**f** Mice were treated with room air or hyperoxia, and DNA fragmentation was measured using a TUNEL assay kit. **g**–**i** MLE15 cells were transfected with control (**g**) or miR-185-5p (**h**) mimics and treated with hyperoxia for 24 h, then necroptotic cell death was measured using a FACS-based necroptosis cell death assay. **g**, **h** The four quadrants of the FACS scattergram represent necroptotic (Q1), late apoptotic (Q2), early apoptotic (Q3), and live (Q4) cells. The figure in panel a represent three independent experiments (*n* = 4 samples per group) with identical results. The figures in (**b**) and (**c**) represent three independent experiments (*n* = 4 mice per group) with identical results. The figure in panels **d**–**i** represent two independent experiments (*n* = 4 mice per group) with identical results. Mean ± SD; *p < 0.05, **p < 0.01.

To determine whether miR-185-5p is released by epithelial cells via EVs after hyperoxia, we subjected mouse alveolar type II cells (MLE15) and wild-type (WT) C57BL/6 mice to hyperoxia (100% oxygen) treatment. Previous screening arrays showed an increase in EV-miR-185 in serum after hyperoxia, and we confirmed this result in our current study. We found that EV-packaged miR-185-5p is significantly induced in a time-dependent manner in MLE15 cell-culture media, BALF, and serum (Fig. 7a–c). Notably, we were unable to detect free miR-185-5p in mouse serum by RT-qPCR. At the same time, we confirmed the epithelial cell death after hyperoxia in vivo. WT C57BL/6 mice were subjected to hyperoxia (100% oxygen) treatment, and then used a TUNEL assay kit was used to detect DNA fragmentation. Significant cell death was detected in mouse lung tissue (Fig. 7d–f). Using a necroptosis cell death assay measured with FACS, we found that treatment of MLE15 cells with hyperoxia and miR-185-5p mimics lead to an upregulation in necroptotic cell death (Fig. 7g–i).

## Discussion

**Fig. 8:**
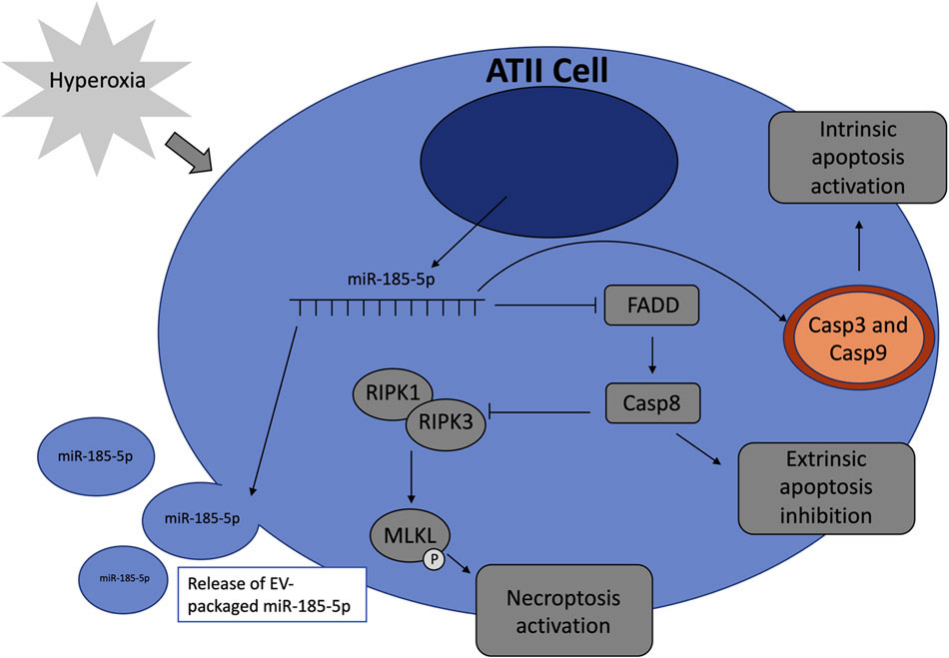
Proposed pathway of necroptosis and apoptosis regulation by hyperoxia-induced miR-185-5p. Schematic of the function of miR-185-5p in alveolar type II cells after hyperoxia.

This paper highlights a novel necroptotic regulating mechanism exerted by miR185-5p, which has not yet been reported. All together, we found that miR-185-5p promotes necroptosis in alveolar type II cells via modulating FADD/caspase-8 pathways. In addition, we showed that miR-185-5p induces apoptosis in ATII cells via promoting caspase 9-mediated intrinsic pathway, despite that it has no effects on caspase 8-associated extrinsic pathway. It is possible miR-185-5p may also have a similar regulatory role in alveolar type I cells, however, our data showed a less significant induction of miR-185-5p in alveolar type I cells in response to hyperoxia, therefore, we focused on alveolar type II cell specifically (Fig. 1a, b) (Fig. 8).This result is consistent with our previous report showing that miR-185 is a cell death regulatory miRNA based on our findings specifically in Beas2B cells, a bronchial epithelial cell line^20^. However, in the setting of ARDS and lung injury, the main focus should be on alveolar epithelial cells, rather than large airway epithelial Beas2B cells. Therefore, in this current work, we focused on ATII cells.

A wide range of regulatory miRNAs, including miR-185, are secreted in the lungs and can be found in both BALF and serum^30,31^. Like many regulatory miRNAs, miR-185-5p can play a significant role in cell death in response to physiological stress^20^. Our results uncover an additional role that miR-185 plays in alveolar epithelial cell death, which is an essential feature in the development of ARDS. Our studies further indicate that miR-185 has the potential to serve as a diagnostic marker for cell death and potentially a therapeutic target in the near future.

Apoptosis can be triggered by either the intrinsic or extrinsic pathway, both of which are involved in HALI. The extrinsic pathway is initiated by the stimulation of Fas receptors which are located on the cell membrane, whereas the intrinsic pathway is initiated by the release of signal factors from the cells mitochondria^4^. In the extrinsic pathway, ligands bind to Fas receptors on the membrane, causing an aggregation of Fas receptors which will recruit FADD^6^. FADD then recruits caspase-8 to form death-inducing signal complex, activating caspase-8 which will subsequently activate caspase-3, leading to cell degradation^4^. The intrinsic pathway can be initiated by mitochondrial stress from stimuli such as DNA damage or heat. In response to mitochondrial stress, two cytoplasmic proapoptotic proteins, BAX and BID, bind to the outer mitochondrial membrane, working with a mitochondrial proapoptotic protein, BAK, promoting the release of cytochrome c from the mitochondria^6^. Cytochrome c then binds to and activates Apaf-1, which allows it to then bind and activate caspase-8^6^. Activated caspase-8 will then activate caspase-3, ultimately leading to cell degradation. In our in vitro model, we found that miR-185-5p induces the intrinsic pathway, but no the extrinsic pathway. Our results showed miR-185-5p seems to inhibit caspase-8, which is associated with the extrinsic pathway (Fig. 5c). This result is consistent as the cytosol miR-185-5p enhances necroptosis via suppressing caspase-8.

Apoptosis is a form of programmed cell death that is mediated by caspases. It plays an essential role in the maintenance of homeostasis and the growth of tissues, as well as in immune response. Necroptosis, a programmed necrosis, emerges as a backup mechanism when apoptosis is non-functional either genetically or pathogenically. It involves the release of intracellular “danger signals” which results in considerable inflammation.

Both FADD and caspase-8 are essential components involving in the extrinsic pathway of apoptosis. On the other hand, FADD and caspase-8 also regulate necroptosis by inhibiting the binding of RIPK1 and RIPK3^27,29^. By blocking the association between RIPK1 and RIPK3, MLKL cannot be subsequently phosphorylated in the final step of necroptosis. Therefore, FADD and caspase-8 play a central role in the crossroad between apoptosis and necroptosis, linking together these two forms of cell death. If levels of FADD and caspase-8 are elevated during cell stress, apoptosis will be activated. MiR-185-5p acts on the FADD/caspase-8 axis during cell stress through inhibition, thus involving both mechanisms and potentially regulating both apoptosis and necroptosis.

Our previous report suggests that miR-185 promotes DNA damage, an intracellular event which certainly promotes intrinsic apoptosis. Our current finding is consistent with our previous report by showing that miR-185-5p activates caspase-9-associated intrinsic pathway and subsequently caspase-3/7. Interestingly, elevated miR-185-5p level suppresses the extrinsic pathway components FADD and caspase-8, suggesting that it potentially switches apoptosis to necroptosis in the setting of prolonged exposure of hyperoxia. By involving in both apoptosis and necroptosis, miR-185-5p potentially is a strong biomarker indicating inevitable alveolar cell death and subsequent inflammatory lung responses in response to prolonged oxidative stress. In addition, EV-cargo miR-185-5p is detectable in BALF and serum, further indicating a potential role as a diagnostic/prognostic marker for ARDS-associated alveolar epithelial cell death.

As mentioned previously, miR-185-5p was unable to be detected by RT-qPCR in mouse serum. Therefore, detection of EV-cargo miR-185-5p is a novel way to develop a biomarker for the diffuse alveolar cell death (DAD) in ARDS. Potentially, future directions can aim to determine the origin of EVs that contain miR-185-5p. Given the ability of EVs to transfer cargo between the origin and target cell, as well as the presence of EVs in BALF and serum, EVs possess the potential to be useful biomarkers of ARDS. Future studies should investigate the possibility to using such approaches in diagnosing acute pulmonary diseases.

Hyperoxia not only induces necroptosis, but also apoptosis and their cross-talk between the two mechanisms of cell death. In addition, both FADD and caspase-8 are known to play a role in the apoptotic pathway as well^32^. Therefore, how miR-185 mediates the crosstalk of apoptosis and necroptosis may require further investigation in the future to acquire a more complete understanding of the role of miR-185 in cell death. MiR-185 probably can be used as a diagnostic marker in the future, but not a therapeutic target as it involves both apoptosis and necroptosis. Future directions should also aim to identify more miRNAs that have synergistic effects with miR-185. Ultimately, we hope to develop a reliable marker using a repertoire of EV-cargo miRNAs. In addition, in the future we should work to develop cell-specific RNA molecule delivery using an EV-mediated manner. This would provide clinicians with an effective method of drug delivery in a variety of diseases and illnesses.

There may be some pitfalls and problems of this study that should be identified and noted. Firstly, HALI is a model mimicking sterile stimuli-associated ARDS.In addition, it should be noted that this study is conducted after hyperoxia, therefore it remains unknown whether the observations in this study can be extrapolated to other stimuli induced oxidative stress. Furthermore, both sterile and infectious stimuli can trigger ARDS. Whether infectious stimuli also leads to elevated miR-185 levels in ATII cells requires further investigation. Lastly, this study used MLE15 and E10 cell lines for in vitro studies as opposed to primary cells isolated from mice.

In conclusion, we found that miR-185-5p promotes alveolar epithelial cell necroptosis by moderating FADD/caspase-8 pathways. This paper potentially uncovers new targets for the development of diagnostic markers by offering a potential detailed novel mechanism for the control of alveolar epithelial cell necroptosis stimulated by hyperoxia-associated ROS.

## Materials and methods

### Animal and cell culture

WT C57BL/6 mice (male, 6–8 week of age) were obtained from Jackson Laboratory (stock #000664, Bar Harbor, ME). All the protocols and methods involving animals in this study were approved by the institutional animal care and use committee (IACUC) of Boston University. All experiments were performed in accordance with relevant guidelines and regulations approved by the IACUC of Boston University.

MLE15 cells were obtained from Whitsett Cincinnati Children Hospital and cultured in DMEM with 10% FBS and 1% penicillin/streptomycin (GIBCO, Grand Island, NY). All cells were cultured at 37 °C in a humidified atmosphere with 5% CO_2_-95% air. For hyperoxia treatment, cells were exposed to hyperoxia (95% oxygen-5% CO_2_) in modular exposure chambers. For miR-185-5p overexpression and inhibition trials, cells grown in a 6-well plate were transfected (Invitrogen™ L3000015) with miR-185-5p mimics, control mimics, or miR-185-5p inhibitors and incubated for 24 h before being treated with hyperoxia. For *N*-acetyl-l-cysteine (NAC) or H_2_O_2_ treatment, 5 mM NAC or 90 μM H_2_O_2_, respectively, was added to the culture medium.

### EV isolation

Previously reported protocols and techniques were applied to isolate EVs. The obtained mouse BALFs, serums, or cell-culture media were centrifuged at 300 g for 5 min to eliminate the inflammatory or dead cells. The supernatant was then collected and centrifuged at 1200 × *g* for 10 min to pellet cell debris. The remaining supernatant was treated with 24% polyethylene glycol (PEG) for a final concentration of 8% PEG, mixed thoroughly by inverting the tubes three times, and left to incubate overnight at 4 degrees Celsius^33^. Precipitated EVs were isolated into a pellet by centrifugation at 1500 × *g* for 30 min at 4 degree Celsius, then the supernatant was removed^33^. All isolated vesicles were re-suspended in PBS.

### RNA preparation, reverse transcription, and quantitative real-time PCR

MiRNeasy Mini Kits (cat. no. 217004; Qiagen, Valencia, CA) were used for purification of total RNA from tissue, cells, and EVs. Single-stranded cDNA was generated according to the manuals of the High-Capacity cDNA Reverse Transcription Kit (cat. no. 4374966, Thermo Fisher Scientific). For miR-185-5p detection, real-time PCR was performed using TaqMan PCR kit (cat. no. 4427975-002271, Thermo Fisher Scientific) and Applied Biosystems StepOnePlus Real-Time PCR Systems (Foster City, CA). The relative miR-185-5p expression level was normalized to mouse *GAPDH*. For the detection of mouse RIPK1, RIPK3, MLKL, FADD, caspase-8, and miR-185-5p, SYBR green-based real-time PCR technique was used as previously described^34^. GAPDH was used as a reference housekeeping gene. List of primers used for qRT-PCR are shown in Table 1.

**Table 1:** Primers used in real-time PCR.

Gene	Sequence (5′-3′)
RIPK1-F	GAAGACAGACCTAGACAGCGG
RIPK1-R	CCAGTAGCTTCACCACTCGAC
RIPK3-F	TCTGTCAAGTTATGGCCTACTGG
RIPK3-R	GGAACACGACTCCGAACCC
FADD-F	GCGCCGACACGATCTACTG
FADD-R	TTACCCGCTCACTCAGACTTC
CASP8-F	TGCTTGGACTACATCCCACAC
CASP8-R	TGCAGTCTAGGAAGTTGACCA
GAPDH-F	ACCACAGTCCATGCCATCAC
GAPDH-R	TCCACCACCCTGTTGCTGTA

### pMLKL and FADD staining and immunofluorescence

MLE15 cells were cultured in a 2 well glass slide (Lab-Tek II Chamber Slide, Thermo Fisher Scientific), transfected with either miR-185-5p mimics/inhibitors or control mimics, and treated with hyperoxia or air for 24 h. After treatment, cells were permeabilized with 4% formaldehyde for 10 min, and washed 3× with PBS. Cells were then incubated with pMLKL primary antibody (ab196436, Abcam) or FADD primary antibody (sc-271748, Santa Cruz) overnight in a 4-degree cooler room. Then, cells were washed with PBS and incubated in fluorescein antibody for 1 h. After nuclear staining and glass slide preparation, pMLKL and FADD immunofluorescence images were captured using a fluorescence microscope (Eclipse TS100, Nikon) at ×20 and ×40 magnification respectively, and analyzed using ImageJ software.

### Western blot analysis

Western Blot analysis was performed as described before^35^. In brief, cells were homogenized in RIPA lysis buffer supplemented with protease inhibitor cocktail and phosphatase inhibitor cocktail (Sigma, St. Louis, MO). Protein lysates were resolved on SDS-PAGE gels before being transferred to the PVDF membrane. Anti-FADD, anti-Caspase-8, anti-RIP, and anti-RIP3 antibodies were purchased from Santa Cruz (sc-271748, sc-56070, sc-133102, and sc-374639 respectively). Mouse monoclonal anti-Actin antibody was used as a loading control. The densities of bands were quantitated using ImageJ software.

### Caspase-3/7 and Caspase-9 activity assay

Caspase-Glo(R) 3/7 and Caspase-Glo(R) 9 Assays (cat. No. G8090 and cat. No. G8210, Promega, Madison, WI) was used for quantification of relative caspase-3/7 and caspase-9 enzyme activity. After treatment of hyperoxia for 1 day, lysis samples were made, and seeded into a white 96-well microplate. Samples were then treated with either Caspase-Glo 3/7 or Caspase-Glo 9 Assay mixture for 30 min, then the luminescence was detected using a microplate reader.

### TUNEL staining and immunofluorescence

TUNEL (terminal deoxynucleotidyl transferase dUTP nick end labeling) staining was performed using a TACS2 TdT DAB kit (Trevigen, Gaithersburg, MD, USA), according to the manufacturer’s instructions for frozen tissue sections. Images were captured using a microscope (Eclipse TS100, Nikon) at ×20 magnification, and ten random fields were examined from each group to determine the average number of TUNEL-positive nuclei in each sample group.

### Cell death by FACS

MLE15 cells were grown in a 6-well plate and transfected (Invitrogen™ L3000015) with miR-185-5p mimics or control mimics, and incubated for 24 h before being treated with air or hyperoxia for 24 h. After treatment cells were scraped off the plate, suspended in PBS, and transferred to 1.5 mL sample tubes. Cells were then incubated overnight with anti-mouse RIP3 and Caspase-3 antibodies (Santa Cruz) at 4 °C in PBS. After washing with PBS and centrifugation (12,000 × *g*, 15 min), the pellets were resuspended in 200 μL of PBS and incubated with FITC- or VioBlue-conjugated secondary antibody for 2 h at room temperature (Introvergen). Cells were then washed, centrifuged (12,000 × *g*, 15 min), resuspended in 400 μL of PBS and analyzed by flow cytometry (MACSQuant Analyzer 10, Miltenyi Biotec); data were analyzed using FlowJo software (Treestar, Inc.). Data were interpreted based on previously published reports naming RIP3+ and Casp3- cells as necroptotic^36,37^.

### Statistical analysis

All data were presented as means ± SD. Comparisons between two groups were performed using a two-tailed unpaired Student’s ***t***-test for statistical significance. *P* < 0.05 was considered statistically significant; *P < 0.05; **P < 0.01

## Data Availability

The data that support the findings of this study are available from the corresponding author, upon reasonable request.
